# Guest editorial: Interventional radiology in India - The road ahead

**DOI:** 10.4103/0971-3026.40300

**Published:** 2008-05

**Authors:** SM Brigadier Chander Mohan

**Affiliations:** Department of Radiology, Military Hospital, Jodhpur - 342 010, India

Interventional radiology (IR) is the ‘medical specialty devoted to advancing patient care through the innovative integration of clinical and imaging-based diagnosis and minimally invasive therapy.’[1] Over the years, there has been a gradual and steady growth in this exciting radiology subspecialty in India. In the seventies and eighties, IR practice was limited to hospitals in New Delhi, Mumbai, Trivandrum, and Lucknow, but over the years there has been an expansion in the practice of IR in India as a whole.

When one looks back at IR's steady growth in India, it is clear that this is not merely an outcome of the growth of diagnostic radiology practices but is due to a combination of factors, which include increasing demands from referring physicians, the widespread availability of imaging equipment for guided procedures, IR's potential to serve as a convenient alternative to open surgical procedures and reduce recovery time, and the tremendous advances in IR hardware.

Today, IR is an integral part of various clinical procedures, finding a role in vascular diseases, oncology, stroke management, women's health, pediatrics, and back pain. Available to us in India are a variety of new IR techniques that have been successfully incorporated into clinical practice, which involve the use of catheters, guide wires, glide wires, balloons for angioplasty, coils, liquid embolizing agents, stents and stent grafts, radiofrequency ablation devices, thrombectomy devices, etc.

Let us briefly look at the leading causes of death worldwide. In high-income countries the list includes heart disease, stroke, lung cancer, colon and rectum cancers, breast cancer, and stomach cancer. In low- and middle-income countries, in addition to the above, the common causes include HIV/AIDS, perinatal conditions, tuberculosis, and road traffic accidents.[2] The clinical management of these varied entities places a burden on society and drains the exchequer. As interventional radiologists, we should encourage the timely and judicious use of IR techniques in the prevention and cure of diseases.

When we look at the future of IR in India, there are two challenges that merit scrutiny and deliberation. The first is the need for vigorous efforts for the motivation of students and radiology residents in all the leading Indian medical institutions so that we can create a second rung of specialists who can gradually step into the shoes of the current practitioners, as is happening in many other countries.[3]

The second challenge is clinical gene therapy. This rapidly developing and promising therapeutic modality is knocking on our doors. Gene therapy involves ‘transferring recombinant genetic material (DNA/ RNA) into the host cell in order to change the gene expression in the host cell to gain a therapeutic effect.’[4] Interventional radiologists will ‘play an important role in the transmission of genetic materials to the target cells,’[5] using percutaneous injection or catheter systems. In the times ahead, interventional radiologists will play an increasingly important role in selecting, guiding, and monitoring clinical gene therapy.[6]

Research in gene therapy applications is underway in diverse areas, including infections, malignancies, metabolic disorders, and enzyme deficiencies. Besides these, the treatment of ischemia of the myocardium and extremities with angiogenetic growth factors to promote collateral artery development is being evaluated. Similarly, the role of gene therapy in treating stenoses following balloon angioplasty or stenting and in therapeutic angiogenesis are also being evaluated.[5]

It is therefore important that interventional radiologists be prepared, continuously updating their knowledge to keep abreast of the advances in molecular imaging and clinical gene therapy applications and issues.[7] The interventional radiologist must know it all: from the awareness of gene therapy terminology to concepts in cloning, vectors, and detection of their expression; from a functional knowledge of molecular imaging and immunology, to understanding the different types of catheters used in the practice of gene transfer technology, such as pressure diffusion, passive diffusion, and mechanical and electrically strengthened catheters.[5] Interventional radiologists should also recognize and be aware of the ongoing advances in the development of novel treatment technologies, commonly used targeted tracers and probes, and of the visualization tools employed to analyze targeted therapy. The time is not far, when catheter-based delivery of tagged stem cells to target lesions becomes an established procedure.

Indeed the situation experienced by practicing interventional radiologists today has been correctly summed up by Brian Stainken: IR “… appeals to people who have a creative streak, people who like procedural care, people who like to use their hands, people who want to make a difference.”[1]

There are exciting times ahead on the IR road with brightly shining milestones along the way. For those interested in participating in IR academic activities, the Indian Society of Vascular and Interventional Radiology (ISVIR) is a useful professional forum. Its website is http://www.isvir.in/
